# Artificial Intelligence-Driven Recommendations and Functional Food Purchases: Understanding Consumer Decision-Making

**DOI:** 10.3390/foods14060976

**Published:** 2025-03-13

**Authors:** Wenxin Wang, Zhiguang Chen, Jiwei Kuang

**Affiliations:** 1 Leeds University Business School, University of Leeds, Leeds LS2 9JT, UK; 2 Panxi Crops Research and Utilization Key Laboratory of Sichuan Province, College of Agricultural Sciences, Xichang University, Xichang 615000, China; 3 Qinghai Tibetan Plateau Key Laboratory of Agricultural Product Processing, Academy of Agriculture and Forestry Sciences, Qinghai University, Xining 810016, China

**Keywords:** AI-driven recommendations, functional foods, purchase intention, AI recommendation personalization, AI recommendation transparency, consumer behavior

## Abstract

Amid rapid advancements in artificial intelligence (AI), personalized recommendation systems have become a key factor shaping consumer decision-making in functional food purchases. However, the influence of AI recommendation characteristics on purchase intention, particularly the underlying mediating mechanisms, remains underexplored. This study aims to investigate how AI recommendation features (personalization and transparency), along with functional food attributes (perceived health benefits and perceived naturalness), influence purchase intention through the mediating roles of perceived packaging and perceived value. Grounded in the Stimulus–Organism–Response framework, data were collected via a structured questionnaire survey, and structural equation modeling was employed for hypothesis testing and model validation. The results indicate that AI recommendation personalization significantly enhances purchase intention both directly and indirectly, while transparency influences purchase intention only through perceived value, emphasizing its role in fostering trust rather than directly driving purchasing behavior. Additionally, perceived health benefits positively influence purchase intention both directly and through mediation, whereas perceived naturalness affects purchase intention only indirectly via perceived value. These findings contribute to consumer behavior research by elucidating psychological mechanisms underlying AI-driven purchase decisions while also providing insights for functional food marketers on how to effectively integrate AI recommendation systems to enhance consumer engagement.

## 1. Introduction

With the growing emphasis on health and wellness, global consumers are increasingly taking control of their health and expecting science-backed solutions to support their well-being. According to McKinsey’s Future of Wellness report in 2024, the wellness market in the United States alone has reached $480 billion, growing at 5 to 10 percent annually, while 20 percent of consumers in the United Kingdom and the United States and 30 percent in China actively seek personalized products and services based on biometric data [1]. These trends underscore not only the rising demand for functional foods but also the growing expectation for personalized health solutions, with AI technologies playing an essential role in delivering tailored recommendations.

Consumers are shifting from merely fulfilling basic nutritional needs to seeking foods that offer additional health benefits. Functional foods, which provide physiological advantages beyond basic nutrition, have gained significant market attention as a means of promoting health and preventing diseases [2]. Unlike conventional foods, functional foods contain bioactive ingredients that support immune function, digestive health, and cardiovascular well-being [3]. For instance, probiotic-rich foods contribute to gut microbiota balance, while omega-3-fortified products have been linked to reduced risks of heart-related conditions. As chronic diseases and aging populations continue to rise globally, functional foods are becoming an essential part of consumer health management.

The increasing demand for functional foods is accompanied by growing challenges in consumer decision-making. Functional food purchases are often driven by health considerations, yet consumers frequently struggle with evaluating product claims, ingredient efficacy, and potential health benefits [4]. As the market expands, consumers face an overwhelming number of choices, many of which involve complex scientific information and unfamiliar terminology. This decision complexity heightens the need for effective tools that help consumers navigate product selection.

At the same time, artificial intelligence (AI) is transforming consumer behavior through digital marketing and personalized recommendation systems. AI-driven recommendations have become an integral component of e-commerce platforms, using vast amounts of consumer data to generate tailored product suggestions [5]. These systems analyze browsing history, purchase behavior, and dietary preferences to improve the relevance of recommendations, reducing consumer search costs and facilitating decision-making. Given that functional foods are typically purchased with specific health objectives in mind, AI recommendation systems play a crucial role in matching consumers with suitable products based on their dietary needs and health goals.

Compared to traditional marketing strategies, which generally rely on mass communication and static promotional content, AI-driven recommendation systems offer real-time, personalized guidance tailored to individual health concerns [6]. This is particularly important in the functional food sector, where consumers often seek products that address highly specific health needs and expect transparent, science-based support in making informed decisions [7]. Furthermore, unlike other digital marketing tools such as banner ads or email campaigns, AI-powered systems actively adapt to user behavior and preferences, continuously refining their suggestions to align with evolving consumer health goals [8]. As a result, AI recommendation systems provide a more dynamic and responsive mechanism to reduce decision complexity and enhance confidence in functional food choices.

Although AI recommendation systems are widely utilized in online retail and digital marketing, research on their role in functional food consumption remains limited. Most existing studies on functional food purchasing behavior have primarily examined the influence of product attributes, such as packaging, taste, and perceived health benefits [9]. Meanwhile, AI-related research has largely focused on consumer trust, user engagement, and the technical aspects of recommendation algorithms. However, the unique value of AI in the context of functional food marketing lies in its ability to simultaneously address the informational challenges of health-related purchases and provide individualized product recommendations, a capability that other digital and traditional marketing approaches lack. This dual function of simplifying complex health information and tailoring product suggestions makes AI a particularly critical tool for supporting consumer decision-making in the wellness and nutrition sectors [10]. Despite this potential, few studies have explored how specific AI recommendation characteristics (e.g., personalization and transparency) interact with health-driven product attributes to influence purchase decisions, particularly in the context of consumer wellness and health management. Furthermore, limited attention has been given to the psychological mechanisms through which these factors jointly shape consumer perceptions and behaviors in online functional food purchasing scenarios.

Meanwhile, although AI technologies have been widely applied across industries such as retail and tourism, their role in the functional food sector is particularly critical due to the unique complexity of health-oriented consumer decision-making [11,12]. Unlike general consumption contexts, functional food purchases often require consumers to assess detailed nutritional information, evaluate scientific health claims, and align product choices with personal health goals [13]. These processes involve higher levels of cognitive effort and health risk assessment, making decision-making more demanding. In such settings, AI recommendation systems offer distinct advantages by delivering tailored, data-driven guidance that simplifies complex health information and provides personalized product suggestions based on individual dietary needs and preferences [14]. This capability is especially valuable in helping consumers navigate the overwhelming array of functional food options and fostering confidence in their choices. Despite the increasing consumer demand for personalized and science-backed health solutions, limited research has examined how AI-driven recommendations address these challenges in the functional food context. Understanding these mechanisms is essential for unlocking the full potential of AI to support informed, health-focused food decisions and for advancing both theory and practice in this emerging area.

This gap is particularly important because functional food choices are not solely influenced by product attributes. Unlike other food categories, consumers face an overwhelming amount of information when selecting functional foods, leading to decision fatigue and uncertainty [3]. In this context, AI-driven recommendation systems serve as an important external influence that can simplify choice-making and enhance decision confidence. Understanding this interaction is crucial for both academia and industry, as it provides insights into how AI-driven marketing strategies shape consumer health-related decision-making. Additionally, it offers practical guidance for businesses on whether and how to integrate AI technologies into their marketing strategies and how to leverage targeted functional food recommendations to enhance consumer preferences and engagement.

To address these gaps, this study develops a conceptual model to examine the combined effects of AI recommendation personalization, AI recommendation transparency, perceived health benefits, and perceived naturalness on consumer purchase intention. It further investigates the mediating roles of perceived packaging and perceived value to reveal the mechanisms through which AI recommendations and product attributes influence purchasing behavior. This study builds upon the Stimulus–Organism–Response (S-O-R) framework to examine how AI recommendation system characteristics and functional food attributes influence consumer purchase intention. The SOR model suggests that external stimuli influence consumer cognitive and emotional responses, which subsequently shape purchasing decisions [15]. In this context, both AI recommendation technology and functional food attributes serve as external stimuli, shaping consumer perceptions of functional foods. Based on these objectives, this study addresses the following two research questions:

Research question 1: How do AI recommendation system characteristics and functional food attributes influence consumer purchase intention?

Research question 2: Do perceived packaging and perceived value mediate the relationships between AI recommendation system characteristics, functional food attributes, and purchase intention?

This study contributes to both academic research and practical applications by integrating AI-driven recommendation systems with functional food marketing. First, this study extends consumer behavior theories by revealing the differential effects of AI recommendation personalization and transparency on purchase intention. While prior research has primarily focused on personalization as a key driver of consumer engagement, this study finds that AI recommendation personalization directly increases purchase intention and further enhances it through perceived packaging and perceived value. In contrast, AI recommendation transparency does not directly influence purchase intention but fosters trust, which enhances perceived value and subsequently drives purchasing decisions. These findings refine the application of the SOR framework, positioning AI personalization as a direct stimulus shaping consumer responses while transparency acts as an indirect enabler of decision-making. From a practical perspective, these insights suggest that functional food brands should prioritize AI-driven personalization strategies by tailoring recommendations to individual health goals. For instance, AI algorithms could analyze consumer dietary patterns to recommend probiotic-rich yogurts for gut health or plant-based protein supplements for fitness enthusiasts. Meanwhile, AI transparency should be leveraged as a trust-building tool rather than a direct sales driver. Functional food companies should provide clear explanations for recommendations, incorporating scientific validation, third-party certifications, and user testimonials to enhance consumer confidence in AI-generated suggestions.

Second, this study contributes to the functional food marketing literature by demonstrating the distinct effects of perceived health benefits and perceived naturalness on purchase intention. While perceived health benefits directly drive purchase decisions and are further reinforced through perceived packaging and perceived value, perceived naturalness does not exert a direct effect but instead influences purchase intention entirely through perceived value. This finding suggests that consumers do not purchase functional foods solely for their natural attributes but rather based on their evaluation of the product’s overall benefits and credibility. In practice, these insights highlight the need for functional food companies to tailor marketing strategies according to product attributes. For health-benefit-focused products, such as omega-3-enriched dairy products or fiber-fortified cereals, companies should emphasize scientific validation on both packaging and AI recommendations to strengthen credibility. Functional food brands can collaborate with nutrition experts and integrate clinical research findings into AI recommendation content to help consumers make informed health decisions. Conversely, for naturally positioned functional foods, such as organic teas or additive-free energy bars, brands should focus on enhancing perceived value rather than relying solely on “100% natural” claims. AI-driven recommendations should highlight the broader benefits of natural ingredients, such as sustainability, ethical sourcing, and long-term health advantages, while packaging should reinforce this message through certifications such as “Organic Certified” or “Minimal Processing”.

Third, this study advances research on the mediating role of perceived packaging in AI-driven consumer decision-making. Unlike prior studies that treat packaging as a static product attribute, this study demonstrates that perceived packaging mediates the effects of AI recommendation personalization and perceived health benefits on purchase intention. This finding underscores the role of packaging as a communication channel that bridges AI-generated recommendations with consumer expectations. From a managerial perspective, functional food brands should align packaging design with AI-generated recommendations to ensure consistency in messaging. For example, for companies producing or selling protein-enriched functional drinks targeted at fitness-conscious consumers, packaging should prominently highlight key health claims such as “Supports Muscle Recovery” or “Endorsed by Nutritionists”. This synergy between AI recommendations and packaging strategies strengthens consumer confidence and enhances purchase motivation.

In summary, this study contributes to the theoretical understanding of AI-driven consumer behavior by integrating technological and product-related attributes within the SOR framework. It demonstrates that AI recommendation personalization directly drives purchase intention, while transparency fosters trust and enhances perceived value. Moreover, it reveals the distinct effects of health benefits and naturalness in shaping purchase behavior and highlights the mediating roles of perceived packaging and perceived value. These findings provide actionable insights for functional food brands seeking to optimize AI-driven marketing strategies, enhance consumer trust, and create value-driven consumer experiences in an increasingly digitalized marketplace.

This paper is structured as follows. First, the conceptual framework is introduced, providing a discussion of the key variables and the development of research hypotheses. Next, the methods detail the research design, measurement instruments, and data collection procedures. The Results section then presents the empirical findings. Finally, the Discussion section provides a summary and interpretation of the key results, followed by theoretical and managerial implications, as well as limitations and directions for future research.

## 2. Conceptual Framework and Hypotheses Development

### 2.1. Conceptual Framework

This study employs the Stimulus–Organism–Response (SOR) theory as the theoretical foundation to examine the factors affecting consumers’ purchase intentions toward functional foods. Originally developed and established by reference [11], this model explains how external environmental stimuli trigger internal psychological states (organism), which then drive behavioral responses. The model has been widely applied in consumer behavior research to analyze the relationships among external influences, cognitive and emotional processes, and subsequent decision-making behaviors [10,12].

The SOR framework was selected for this study because of its ability to comprehensively explain how external environmental stimuli influence internal cognitive and emotional processes, which subsequently drive behavioral responses, and is commonly used in online consumer behavior [13,14]. This makes it particularly suitable for the context of functional food consumption, where consumers must navigate complex decisions involving both technological factors and product attributes [15]. Compared to other commonly used models, such as the Technology Acceptance Model (TAM), which primarily focuses on consumers’ perceptions of technology usefulness and ease of use, SOR offers a broader perspective [16]. While TAM is effective in explaining technology adoption behaviors, it is limited in its capacity to account for product-related attributes and the more comprehensive decision-making processes that involve emotional and cognitive reactions to multiple external stimuli. Given that this study examines both AI-driven recommendation characteristics and the intrinsic attributes of functional foods, TAM does not provide a sufficiently robust theoretical foundation to address these combined effects. In contrast, the SOR framework enables the simultaneous analysis of diverse external stimuli and their psychological and behavioral outcomes [13]. Therefore, SOR provides a theoretically appropriate and comprehensive foundation for understanding the multi-dimensional factors that influence consumer decision-making in AI-assisted, health-oriented food purchasing contexts.

Acknowledging that other factors, such as individual differences like age and familiarity with technology, may also influence consumer decisions [13,17], although these are beyond the scope of the present study, which focuses on examining the effects of AI recommendation characteristics and functional food attributes on purchase intention within the SOR framework.

In this study, external stimuli include technological and product-related factors. The technological factors include AI recommendation personalization and AI recommendation transparency, both of which significantly impact consumer trust and decision-making [18,19]. AI recommendation personalization refers to the extent to which AI-driven systems provide tailored product suggestions that align with consumer preferences and health goals [20,21]. This feature enhances user engagement and optimizes purchase decision-making by delivering relevant product recommendations [22,23]. AI recommendation transparency reflects the degree to which AI systems disclose information about the logic, data sources, and processes behind their recommendations [24,25]. Higher transparency increases consumer trust, reduces uncertainty, and strengthens perceptions of fairness, especially in health-related food purchases [26,27].

The product-related factors examined in this study are perceived health benefits and perceived naturalness. Perceived health benefits refer to how much consumers believe a functional food can improve their health, such as boosting immunity, supporting heart health, or aiding digestion [28,29]. Functional foods contain bioactive ingredients like probiotics, omega-3 fatty acids, and dietary fiber, making health benefits a key factor in purchasing decisions [2,30]. Perceived naturalness reflects whether consumers see a product as being made from natural ingredients and free from artificial additives or heavy processing [31,32]. Consumers often view natural products as healthier, safer, and of better quality, which affects their buying behavior [33].

The four external stimuli in this study, comprising two technological factors (AI recommendation personalization and AI recommendation transparency) and two product-related attributes (perceived health benefits and perceived naturalness), were selected due to their central importance in shaping consumer decision-making within the functional food context. Previous research has consistently demonstrated that personalization and transparency are critical elements in building consumer trust and engagement with AI recommendation systems [17,25]. At the same time, perceived health benefits and perceived naturalness are widely recognized as key drivers of consumer acceptance of functional foods [25,34]. Focusing on these variables allows the model to capture the key technological and product-related factors identified in previous studies as influential in shaping purchase intention, thereby aligning with the structure of the SOR framework and supporting theoretical consistency.

These external stimuli influence internal psychological evaluations, which are captured through two mediating variables: perceived packaging and perceived value. Perceived packaging refers to consumers’ subjective evaluation of a product’s packaging, including its design, labeling, functionality, and ability to convey essential product information [35]. In the functional food industry, packaging plays a vital role in enhancing consumer trust and purchase intention by communicating health claims, nutritional content, and certification marks [36]. Research suggests that well-designed packaging can positively impact product credibility, consumer attention, and overall purchasing decisions [37,38]. Perceived value, on the other hand, represents the trade-off consumers make between a product’s benefits (e.g., health and safety) and its costs (e.g., price, effort required to obtain the product) [39]. In the context of functional foods, perceived value extends beyond price to include consumers’ evaluation of a product’s health benefits, safety, and alignment with their personal health management goals [40]. Prior research suggests that functional foods with strong perceived health benefits and credibility-enhancing packaging designs tend to be viewed as higher in value, thereby increasing purchase intentions [41].

Finally, the behavioral response in this study is purchase intention, which represents a consumer’s likelihood of buying a functional food product in the future [42]. Purchase intention is a key predictor of actual purchasing behavior and is influenced by both external stimuli (AI recommendations and functional food attributes) and internal psychological evaluations (perceived packaging and perceived value) [43,44]. In the AI-driven digital food marketplace, personalized recommendations and transparent marketing strategies have been shown to enhance consumer confidence, strengthen product perception, and ultimately drive higher purchase intentions [9].

**Figure 1:**
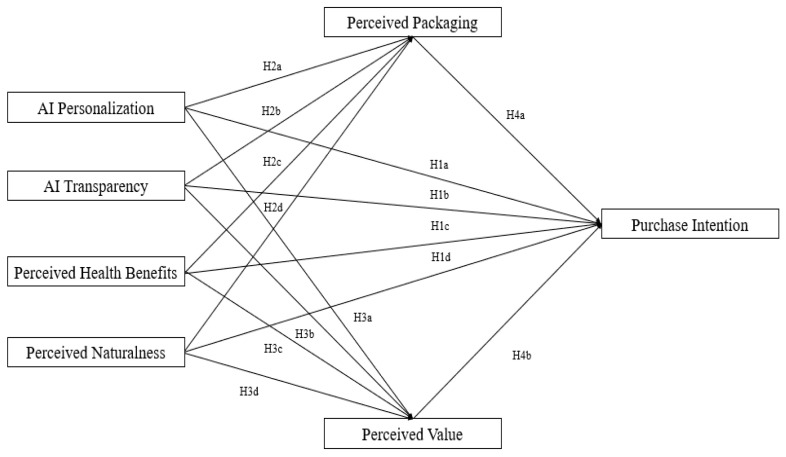
Conceptual Framework.

In summary, this study examines how AI recommendation personalization and transparency, along with functional food attributes such as perceived health benefits and perceived naturalness, affect purchase intention. It also explores the mediating roles of perceived packaging and perceived value in these relationships. Figure 1 presents the conceptual model guiding this research.

### 2.2. Hypotheses Development

#### 2.2.1. Direct Effects of AI Recommendation and Functional Food Attributes on Purchase Intention

According to the S-O-R model, external stimuli trigger internal psychological responses, which subsequently influence consumer behavior [11]. In this study, technological factors represented by AI recommendation system features (personalization and transparency) and product-related factors represented by key attributes of functional foods (perceived health benefits and perceived naturalness) serve as critical external stimuli that shape consumers’ purchase intentions toward functional foods.

With the rapid advancement of digital technologies, personalization and transparency in AI recommendation systems have become critical factors influencing consumer behavior [23]. Personalization enables AI systems to tailor recommendations based on individual consumer interests, preferences, and behavioral data, enhancing both the shopping experience and consumer satisfaction [45]. By leveraging user data such as clickstreams and purchase histories, AI recommendation systems can dynamically adjust their strategies in real time, leading to higher conversion rates and more effective consumer engagement. For example, companies like Amazon and Google Assistant utilize AI-based platforms, such as Amazon Lex, to deliver highly personalized recommendations, which have been shown to positively influence consumer attitudes and increase engagement with products [45]. In the context of nutrition and health, personalized AI-driven recommendation systems have been found to play a key role in shaping consumer behavior by providing targeted suggestions that align with individual dietary needs [46]. In AI-powered environments, personalized recommendations help mitigate information overload, allowing consumers to quickly identify products that meet their specific needs, thereby increasing the likelihood of purchase [23]. Moreover, personalization increases perceived relevance and reduces perceived effort during the decision process, which psychologically primes consumers to attribute greater attention and importance to packaging elements [47]. When recommendations match individual needs, consumers may be more inclined to interpret packaging as supportive evidence reinforcing the suitability and credibility of the product, thereby enhancing their evaluation of packaging quality and informativeness [48].

Transparency, on the other hand, is crucial for building consumer trust in AI recommendation systems. It involves clearly communicating the rationale, data sources, and algorithms behind the recommendations, which helps reduce consumer uncertainty and fosters a sense of reliability [26]. Recent research indicates that transparent recommendation systems effectively alleviate consumer concerns related to data privacy and security, thereby enhancing purchase intentions [49]. For example, when AI systems explicitly explain how recommendations are generated based on users’ past behaviors and preferences, consumer trust in the system significantly increases. This factor is particularly important in the functional food sector, where health-related attributes are closely scrutinized by consumers. Transparency in product recommendations can reduce perceived risks associated with health-related decisions [50] and enhance consumer confidence in the quality and safety of functional foods [51]. By helping consumers understand the health benefits and value of the recommended products, transparency further strengthens their confidence in making purchasing decisions related to functional foods.

In addition to technological factors, product-related attributes such as perceived health benefits and perceived naturalness also play a crucial role in influencing consumer purchase intentions. These attributes act as external stimuli that trigger cognitive and emotional evaluations, ultimately shaping purchasing behavior.

In today’s health-conscious society, concerns about well-being have become a central factor influencing food choices [52]. Functional foods are particularly valued for their potential to promote health and prevent disease [53]. Perceived health benefits serve as a key stimulus, directly affecting consumers’ internal evaluations regarding product value and trust. Functional foods often contain bioactive components, such as probiotics, omega-3 fatty acids, and plant sterols, which are believed to support specific health outcomes like improved digestive health, cardiovascular function, and immune response [54,55]. When consumers perceive that these products can address their health concerns or contribute to their overall well-being, this positive evaluation enhances their likelihood of purchase [56].

Moreover, the perception of health benefits fosters consumer trust, reducing perceived risks associated with purchasing new or unfamiliar food products. Functional foods backed by credible health claims, scientific evidence, and regulatory approvals provide reassurance to consumers, serving as reliable stimuli that trigger positive internal responses such as confidence and security [57]. This trust effectively reduces uncertainty and increases the intention to purchase [55].

Perceived naturalness functions as another critical external stimulus, influencing consumers’ cognitive evaluations of product quality and safety. Naturalness is often associated with food safety, authenticity, and purity [58]. As concerns about artificial additives, synthetic ingredients, and highly processed foods continue to rise, consumers increasingly favor products perceived as natural [59]. Naturalness implies minimal processing and the absence of artificial substances, reducing anxiety about potential health risks and fostering positive emotional responses such as satisfaction and peace of mind.

In the case of functional foods, naturalness also enhances perceptions of product efficacy and quality [54]. Consumers often believe that natural ingredients contribute to superior nutritional value and health outcomes [60]. This belief strengthens internal evaluations of the product, reinforcing both cognitive assessments of quality and emotional attachment, which in turn drive stronger purchase intentions.

Based on the above discussion, the following hypotheses are proposed:

**Hypothesis 1a:**  *AI recommendation personalization positively influences consumers’ purchase intention of functional foods.*

**Hypothesis 1b:**  *AI recommendation transparency positively influences consumers’ purchase intention of functional foods.*

**Hypothesis 1c:**  *Perceived health benefits positively influence consumers’ purchase intention of functional foods.*

**Hypothesis 1d:**  *Perceived naturalness of functional foods positively influences consumers’ purchase intention.*

#### 2.2.2. The Mediating Role of Perceived Packaging

From a consumer psychology perspective, AI-driven personalized recommendations enhance the relevance of product information by analyzing consumers’ historical behaviors, preferences, and needs [61]. This targeted approach not only fulfills consumers’ expectations for personalized experiences but also reinforces their self-identification with the recommended products [21]. When consumers perceive a strong alignment between their preferences and the recommended functional foods, this positive association may extend to their evaluation of the product’s packaging. Drawing from information processing theory, consumers are increasingly exposed to vast amounts of information, which can lead to cognitive overload and difficulty in decision-making [14]. Personalized AI recommendations mitigate this burden by presenting only the most relevant options [23]. As a result, consumers are more likely to focus on packaging as an additional informational cue, leading to a more favorable evaluation of its design, branding, and functional claims. Moreover, personalization increases perceived relevance and reduces perceived effort during the decision process, which psychologically primes consumers to attribute greater attention and importance to packaging elements. When recommendations match individual needs, consumers may be more inclined to interpret packaging as supportive evidence reinforcing the suitability and credibility of the product, thereby enhancing their evaluation of packaging quality and informativeness.

Transparency in AI recommendation systems also plays a critical role in shaping consumer perceptions of product packaging. Transparent recommendation systems explicitly communicate how recommendations are generated, including the underlying data sources and algorithmic mechanisms [62]. This openness fosters trust in both the recommendation system and the associated products. In the context of functional foods, where product credibility and safety are key concerns, transparency reduces perceived risks and strengthens consumer confidence in the product claims and branding elements displayed on the packaging [63]. When consumers perceive the recommendation process as reliable and unbiased, they are more likely to view the product’s packaging as a credible representation of its attributes, further enhancing their positive evaluation. From a psychological perspective, transparency not only fosters trust but also enhances consumers’ perceived control over their purchase decisions by clearly disclosing how recommendations are generated and why specific products are suggested [64]. This greater sense of control reduces feelings of manipulation and empowers consumers to actively engage with the information presented [65]. As a result, consumers may perceive the packaging not merely as a passive display of product information but as an integral, supportive element that aligns with the transparent logic of the AI system. This increased empowerment encourages deeper processing of packaging details, such as health certifications, ingredient lists, and sustainability claims, reinforcing positive evaluations of the product’s authenticity and credibility.

From the perspective of the health belief model, consumers’ perceptions of health benefits significantly shape their product evaluations and purchase intentions [66]. Functional foods are typically marketed based on their health benefits, and consumers who perceive these benefits as credible are more likely to develop favorable evaluations of product packaging. Research suggests that products with strong health claims are often associated with well-designed, professional packaging that effectively conveys these attributes [67]. Packaging elements such as clear nutritional labeling, official health certifications, and endorsements from scientific authorities reinforce the credibility of functional foods, leading to more favorable perceptions.

Perceived naturalness also plays a crucial role in shaping consumer expectations of packaging design. Research in environmental psychology suggests that as sustainability concerns grow, consumers increasingly associate naturalness with eco-friendly and minimally processed products [33]. When consumers perceive functional foods as natural, they expect the packaging to align with sustainability principles, such as the use of biodegradable materials, minimalist designs, and nature-inspired aesthetics [54]. Drawing from cognitive heuristics theory, consumers often rely on simple cues to make inferences about overall product quality [68,69]. In the functional food sector, packaging serves as a key heuristic, influencing perceptions of authenticity and trustworthiness [70]. A well-designed, natural-looking package strengthens consumers’ trust in the product’s ingredients and production process, thereby reinforcing its perceived value.

Based on the above discussion, the following hypotheses are proposed:

**Hypothesis 2a:**  *AI recommendation personalization positively influences consumers’ perception of packaging.*

**Hypothesis 2b:**  *AI recommendation transparency positively influences consumers’ perception of packaging.*

**Hypothesis 2c:**  *Perceived health benefits positively influence consumers’ perception of packaging.*

**Hypothesis 2d:**  *Perceived naturalness of functional foods positively influences consumers’ perception of packaging.*

While personalization and transparency are generally expected to enhance consumers’ evaluations of packaging by increasing relevance, trust, and informativeness, it is important to acknowledge that these effects may not be uniform across all consumer segments. Prior research indicates that certain consumers may experience skepticism toward algorithmic recommendations, perceiving them as intrusive or manipulative, which could dampen the positive influence of personalization and transparency on packaging perception [66]. Additionally, other factors such as age, digital literacy, and cultural attitudes toward technology may moderate how consumers interpret AI-driven product presentations [65]. Future studies could further explore these boundary conditions to deepen understanding of the contexts in which AI-driven packaging perceptions are most effective.

#### 2.2.3. The Mediating Role of Perceived Value

According to the S-O-R model, external stimuli, such as technological and product-related factors, influence consumers’ internal psychological states, which subsequently shape their behavioral responses [11]. In this study, perceived value is regarded as a central component of the internal evaluation process (organism), serving as a mediating factor that links external stimuli to consumer purchase intentions.

In the context of digital consumption, AI technology has facilitated the delivery of personalized recommendation services tailored to consumers’ preferences, purchase histories, and behavioral data [61,71]. Personalized AI-driven recommendations help consumers efficiently navigate a wide range of functional food options, aligning products with their specific health goals and preferences. This precise alignment reduces cognitive effort and minimizes the time required for information searches, enhancing the convenience and efficiency of the purchasing process. As a result, consumers perceive greater value in the recommended products [22]. Furthermore, the data-driven nature of AI recommendations fosters consumer trust. Since these recommendations are based on sophisticated algorithms and large datasets, consumers often perceive them as objective, reliable, and reflective of expert insights [72], which further enhances the perceived value of functional foods. Beyond convenience, personalization contributes to perceived value by reinforcing a sense of relevance and individual fit. When consumers receive recommendations that closely match their unique health needs and dietary goals, they are more likely to perceive the products as thoughtfully selected and highly suitable [73]. This perceived alignment increases satisfaction and strengthens the belief that the products offer superior benefits relative to alternatives, thereby enhancing overall value perception.

Transparency within AI recommendation systems is another critical factor influencing perceived value. Transparency refers to the extent to which consumers can understand how recommendations are generated, including the algorithms and data sources used [26]. Transparent systems provide clear explanations of why specific products are recommended, which helps reduce consumer uncertainty and builds trust in the recommendation process [62]. When consumers feel informed and understand the reasoning behind recommendations, they are more likely to perceive the suggested products as credible and valuable. Moreover, transparency enhances the overall customer experience by fostering a sense of control and reducing perceived risks associated with purchasing decisions. This increased confidence and satisfaction contribute to a higher perceived value of functional foods [26]. From a psychological perspective, transparency not only reduces uncertainty but also increases perceived fairness and informational empowerment [74]. When consumers are aware of how and why recommendations are made, they feel more in control of their decision-making process [75]. This heightened sense of control alleviates concerns about potential bias or manipulation, reinforcing the perception that the recommended products are both credible and tailored to their genuine needs. As a result, transparency strengthens the perceived value by validating the appropriateness and reliability of the functional food options presented.

Perceived health benefits, as a core attribute of functional foods, play a significant role in shaping consumers’ perceptions of value. Functional foods are often marketed based on scientifically supported health claims, such as improving immune function, supporting heart health, or enhancing digestive well-being [29,30]. When consumers perceive these health benefits, they view functional foods not just as sources of basic nutrition but as valuable investments in long-term health. This perception of added health value often justifies higher price points and enhances the overall evaluation of product worth. Additionally, the potential for long-term health benefits—such as reduced medical expenses and improved quality of life—reinforces the perceived value, making functional foods more attractive even when priced at a premium [2].

Similarly, perceived naturalness significantly influences the perceived value of functional foods. Naturalness is often associated with product safety, purity, and minimal processing, attributes that resonate strongly with health-conscious consumers [33]. Consumers who perceive a product as natural tend to believe it contains fewer artificial additives and is produced using high-quality, unprocessed ingredients, which positively affects their evaluation of the product’s quality [54]. Beyond health considerations, naturalness also aligns with broader consumer values related to sustainability and environmental consciousness. For many consumers, choosing natural functional foods reflects a commitment to a healthier lifestyle and eco-friendly practices [76]. This alignment with personal values and lifestyle preferences further enhances the perceived value of these products, increasing their appeal in the marketplace.

Based on the above discussion, the following hypotheses are proposed:

**Hypothesis 3a:**  *AI recommendation personalization positively influences consumers’ perceived value of functional foods.*

**Hypothesis 3b:**  *AI recommendation transparency positively influences consumers’ perceived value of functional foods.*

**Hypothesis 3c:**  *Perceived health benefits positively influence consumers’ perceived value of functional foods.*

**Hypothesis 3d:**  *Perceived naturalness of functional foods positively influences consumers’ perceived value.*

#### 2.2.4. The Influence of Mediating Variables on Purchase Intention

In the modern consumer market, packaging functions not only as a protective layer but also as a critical medium for conveying product information and shaping brand perception [68]. This is particularly relevant in the functional food sector, where packaging serves as an essential channel for communicating key attributes such as health benefits, ingredient composition, and usage instructions. Given that consumers often encounter a product’s packaging before engaging with its detailed information, effective packaging design can significantly influence their evaluation of the product. Clear, concise, and visually appealing packaging facilitates consumer comprehension, reduces search costs, and simplifies decision-making. Research suggests that while consumers prioritize the health effects of functional foods, they also consider extrinsic attributes, including packaging elements, in their purchasing decisions [76].

Beyond information dissemination, packaging design can enhance product appeal and contribute to a positive consumer experience. Empirical studies indicate that innovative packaging features, such as eco-friendly materials and distinctive designs, positively influence purchase intentions in the food sector [72]. In a competitive market, packaging differentiation can serve as a strategic advantage by capturing consumer attention and fostering brand recognition [77]. For example, the use of sustainable materials, such as biodegradable or recyclable packaging, aligns with the growing consumer preference for environmentally responsible products. Additionally, user-friendly packaging designs, such as resealable or portable formats, improve convenience and functionality, further reinforcing purchase intentions.

Perceived value is a fundamental determinant of consumer purchase intention, particularly in the functional food sector [78]. Consumers assess product value by weighing intrinsic attributes, such as health benefits and naturalness, against extrinsic factors, including price fairness and brand credibility [79]. When functional foods are perceived as offering high value, consumers are more likely to trust their quality, efficacy, and safety, which in turn increases their willingness to purchase.

Trust plays a pivotal role in the relationship between perceived value and purchase intention. Functional foods that are backed by scientific validation, made from high-quality natural ingredients, or associated with well-established brands tend to be perceived as more credible [79]. This heightened credibility mitigates perceived risks, thereby reinforcing consumer confidence in the purchase decision. For instance, when a functional food product highlights its use of clinically tested ingredients and its endorsement by a reputable brand, consumers are more likely to perceive it as reliable and beneficial, strengthening their intention to purchase.

Based on the above discussion, the following hypotheses are proposed:

**Hypothesis 4a:**  *Perceived packaging of functional foods positively influences consumers’ purchase intention.*

**Hypothesis 4b:**  *Perceived value of functional foods positively influences consumers’ purchase intention.*

## 3. Materials and Methods

### 3.1. Research Design

This study adopts a quantitative research approach, utilizing a questionnaire survey to examine how AI recommendation system characteristics (personalization and transparency) and functional food attributes (perceived health benefits and perceived naturalness) influence consumers’ purchase intentions. To ensure the validity and reliability of the measurement instruments, the questionnaire underwent multiple rounds of expert evaluation. A panel of scholars specializing in food science, marketing, and consumer behavior systematically reviewed the survey structure, measurement scales, and item formulations. Their feedback was incorporated to refine the clarity, coherence, and construct validity of the instrument. Before the formal survey, a small-scale pretest (*n* = 30) was conducted to assess the clarity, wording, and comprehensibility of the questionnaire items, particularly the newly developed items. Based on the feedback received, minor revisions were made to improve item expressions and enhance the overall readability of the survey instrument. This process further supported the content validity of the measures and ensured their suitability for large-scale data collection.

The final questionnaire comprised two sections. The first section collects demographic information (e.g., gender, age, education level) to ensure sample diversity. The second section includes measurement items for the study’s key variables, all evaluated on a 7-point Likert scale (1 = strongly disagree, 7 = strongly agree). Data were collected through a convenience sampling method, commonly used in consumer behavior research, to efficiently gather diverse responses from relevant participant groups [80]. The data were collected online across China, with project coordination based in Xichang. Participants were recruited via online platforms, targeting individuals with prior experience purchasing functional foods through digital channels and receiving personalized product suggestions (e.g., online shopping platforms or health-related applications). While convenience sampling may limit generalizability to some extent, the sample reflects a broad demographic distribution, supporting the validity of the findings within the study context [80].

To ensure data quality and minimize the risk of duplicate or invalid responses, multiple control measures were applied during data collection and cleaning. These included IP address checks to identify potential duplicates, monitoring completion times to detect abnormally rapid submissions, and removing responses with identical answer patterns or other indicators of low-quality data. After these procedures, 407 valid responses were retained from the 420 collected questionnaires for subsequent analysis. The adequacy of the sample size was carefully evaluated in relation to the model complexity. The final dataset includes 7 latent variables and 42 observed variables, meeting commonly recommended thresholds for structural equation modeling (SEM). Previous studies suggest that a minimum ratio of 10 cases per estimated parameter is necessary to ensure stable model estimation [80,81], while others recommend a minimum of 200 cases for complex SEM models [82]. Recent research further supports these guidelines [83]. Accordingly, with 407 valid responses, the sample size in this study is considered sufficient, ensuring the robustness and reliability of the SEM analysis.

### 3.2. Measures

This study examines four independent variables (AI recommendation personalization, AI recommendation transparency, perceived health benefits, and perceived naturalness), two mediating variables (perceived packaging and perceived value), and one dependent variable (purchase intention). The conceptual definitions of these variables have been discussed in the conceptual framework. To ensure content validity, measurement scales were primarily adapted from well-established studies, with modifications made to align with the context of functional food consumption and AI-driven recommendations. In cases where no directly applicable scales were available, new items were developed based on the relevant literature and theoretical frameworks. All constructs were measured using a 7-point Likert scale (1 = strongly disagree, 7 = strongly agree).

AI recommendation personalization and AI recommendation transparency capture key technological aspects of AI-driven marketing systems. AI recommendation personalization was measured using items adapted from reference [18], assessing the extent to which AI systems provide tailored recommendations that align with consumers’ personal preferences and health goals. A representative item is “The AI-recommended functional foods match my personal dietary preferences and health goals”. AI recommendation transparency was assessed based on the frameworks [19], evaluating the clarity with which AI systems disclose information about their recommendation processes, including data sources and decision-making criteria. A sample item includes “The AI system provides clear explanations about the health benefits of the recommended functional foods, helping me understand its recommendations”.

Perceived health benefits and perceived naturalness reflect consumers’ evaluations of functional food attributes. Perceived health benefits were measured using items adapted from reference [28], capturing the extent to which consumers believe functional foods contribute to improved health outcomes. A sample item is “I believe that functional foods can significantly improve my health”. Perceived naturalness, assessed based on established constructs in food consumer research, measures consumer perceptions regarding the natural composition of functional foods, particularly the presence of natural ingredients and the absence of artificial additives. A representative item is “This functional food is primarily made from natural ingredients rather than synthetic substances”.

The mediating variables, perceived packaging and perceived value, were included to explore the indirect effects of AI recommendation system characteristics and functional food attributes on purchase intention. Perceived packaging was measured to capture consumers’ evaluations of packaging design, emphasizing its attractiveness and functional qualities. A representative item is “The packaging design of this functional food attracts my attention”. Perceived value was measured following reference [30], encompassing consumers’ overall assessment of a product’s worth, including health-related benefits and a sense of personal responsibility for health management. A sample item is “I feel responsible for maintaining my health through healthy food choices”.

The dependent variable, purchase intention, was measured using items adapted from the literature [84], assessing consumers’ likelihood of purchasing functional foods in the future. A typical item includes “I intend to purchase functional foods in the future”.

By adopting measurement scales grounded in prior research while tailoring them to the study context, this research ensures a robust evaluation of key constructs. The integration of validated scales with necessary refinements strengthens the reliability and validity of the measurement model, contributing to the rigor of this study’s empirical framework.

### 3.3. Data Analysis Methods

This study employed structural equation modeling (SEM) to test the proposed hypotheses and examine the relationships among variables. SEM is a multivariate statistical technique that allows for the simultaneous estimation of multiple relationships while accounting for measurement errors. Compared to traditional regression analysis, SEM is particularly suitable for studies involving latent constructs, such as AI recommendation system characteristics and the perceived value of functional foods, which cannot be directly observed and require multiple indicators for accurate measurement [85]. This study employed SEM to test the proposed hypotheses and examine the relationships among variables. SEM is a multivariate statistical technique that allows for the simultaneous estimation of multiple relationships while accounting for measurement errors. Compared to traditional regression analysis, SEM is particularly suitable for studies involving latent constructs, such as AI recommendation system characteristics and the perceived value of functional foods, which cannot be directly observed and require multiple indicators for accurate measurement [85].

The SEM procedure in this study consists of two main components: the measurement model and the structural model. The measurement model assesses the reliability and validity of latent constructs, while the structural model tests the hypothesized relationships between variables. Since some measurement scales were newly developed for this study, Exploratory Factor Analysis (EFA) was first conducted to confirm the factor structure before performing Confirmatory Factor Analysis (CFA) to assess the reliability and validity of the constructs.

To ensure the robustness of the measurement scales, EFA was conducted for all constructs. The Kaiser–Meyer–Olkin (KMO) test was used to assess sampling adequacy, and Bartlett’s test of sphericity was applied to determine whether the data were suitable for factor analysis. A KMO value greater than 0.6 and a significant Bartlett’s test (*p* < 0.05) indicate that the dataset is appropriate for factor extraction [85]. Principal component analysis (PCA) with Varimax rotation was employed to extract factors, ensuring that each retained factor explained a sufficient proportion of variance. Factors were retained based on eigenvalues greater than 1, a cumulative variance explanation exceeding 60%, and factor loadings above 0.5.

Following EFA, CFA was performed to validate the measurement model. Convergent validity was assessed using standardized factor loadings, Average Variance Extracted (AVE), and Composite Reliability (CR), with AVE values exceeding 0.5 and CR values above 0.7 indicating acceptable convergent validity. Discriminant validity was examined using the Fornell–Larcker criterion, which requires that the square root of each construct’s AVE be greater than its correlations with other constructs, confirming adequate discriminant validity [85].

The structural model was developed to test the hypothesized relationships between AI recommendation characteristics, functional food attributes, and purchase intention. Maximum Likelihood Estimation (MLE) was employed for parameter estimation, and the significance of mediation effects was tested using the Bootstrap method with 5000 resampling iterations and a 95% confidence interval (CI). Model fit was assessed using multiple indices, including absolute fit indices (χ²/df, RMSEA, SRMR) and incremental fit indices (CFI, TLI). These indices collectively provide a comprehensive evaluation of the model’s fit and the robustness of the findings.

### 3.4. Data Analysis Procedure

Data analysis was conducted using AMOS 23.0, following a systematic approach to ensure rigor and validity. First, data preprocessing and descriptive analysis were performed, including data cleaning to remove incomplete or inconsistent responses and computing descriptive statistics (means, standard deviations, and correlation coefficients) to examine variable distributions. Second, EFA was conducted to assess the factor structure and ensure alignment with theoretical expectations. The suitability of the data for factor analysis was evaluated using the KMO test and Bartlett’s test of sphericity, and factors were extracted using principal component analysis with varimax rotation. Third, CFA was conducted to validate the measurement model, assessing reliability and validity. Fourth, structural model estimation and path analysis were performed to test the hypothesized relationships, estimate direct and indirect effects using MLE, and assess mediation effects via Bootstrapping with 5000 iterations. This rigorous analytical procedure ensures the robustness of the findings and provides strong empirical support for the study’s conclusions.

## 4. Results

### 4.1. Descriptive Statistics

The sample consisted of 407 respondents, retained after rigorous data cleaning procedures to ensure high data quality and the removal of incomplete or duplicate responses. The sample presented a nearly equal gender distribution (51.35% male, 48.65% female). The majority of participants (50.61%) were aged 25–34 years, followed by those aged 35–44 years (24.08%), 18–24 years (13.51%), and 45 years and above (11.79%). Regarding educational background, the majority held a bachelor’s degree (52.09%, n = 212), followed by participants with a master’s degree (22.60%, n = 92), high school education or below (21.87%, n = 89), and doctoral degrees (3.44%, n = 14). These distributions reflect a diverse sample across gender, age, and education levels, providing a robust basis for subsequent analyses. Moreover, the demographic characteristics suggest a reasonable representation of consumer groups that are active in digital environments and may have experience with online functional food purchases, supporting the applicability of the findings within this context.

### 4.2. Measurement Model Evaluation

#### 4.2.1. Reliability and Convergent Validity

This study conducted EFA on the constructs. The KMO test yielded a value above 0.6, and Bartlett’s test of sphericity was statistically significant (*p* < 0.05), confirming the suitability of the data for factor analysis. The results further indicated that the cumulative variance explained exceeded 60%, and all measurement items exhibited factor loadings above 0.5, aligning with the expected factor structure. These findings confirm that the measurement scales demonstrate satisfactory structural validity for this study. Internal consistency was assessed using Cronbach’s alpha (α), with Cronbach’s α ranging from 0.743 to 0.823, indicating acceptable to good reliability and ensuring the measurement scales were internally consistent.

Convergent validity was evaluated using AVE and CR. As shown in Table 1, all AVE values met or exceeded the 0.5 threshold, while all CR values surpassed 0.7, confirming satisfactory convergent validity [86]. Standardized factor loadings were also within the acceptable range, further supporting the measurement model’s validity. These results indicate a robust measurement model, confirming its suitability for SEM.

#### 4.2.2. Discriminant Validity

Discriminant validity was assessed using the Fornell–Larcker criterion, which ensures that each construct is empirically distinct within the theoretical model. This approach compares the square root of each construct’s AVE with its inter-construct correlations [86]. The results confirm that the square root of each construct’s AVE exceeded its corresponding inter-construct correlations, demonstrating satisfactory discriminant validity.

### 4.3. Correlation Analysis

Correlation analysis was conducted to examine the relationships among key constructs in the model. Table 2 shows the correlation matrix. The results indicate significant positive correlations between purchase intention (PI) and its key predictors. Specifically, PI is strongly associated with the perceived value of functional foods (PV) (r = 0.507, *p* < 0.05) and perceived packaging of functional foods (PPF) (r = 0.422, *p* < 0.05). Additionally, PI exhibits positive correlations with AI recommendation personalization (PLAR) (r = 0.447, *p* < 0.05), perceived health benefits of functional foods (PHB) (r = 0.424, *p* < 0.05), AI recommendation transparency (TAR) (r = 0.282, *p* < 0.05), and perceived naturalness of functional foods (FFA) (r = 0.354, *p* < 0.05). These findings reinforce the central role of purchase intention in the theoretical model.

Furthermore, significant positive correlations are observed between PPF and key predictors, including PLAR (r = 0.365, *p* < 0.05), PHB (r = 0.376, *p* < 0.05), FFA (r = 0.311, *p* < 0.05), and TAR (r = 0.269, *p* < 0.05). Similarly, perceived value (PV) is positively correlated with PLAR (r = 0.439, *p* < 0.05), PHB (r = 0.404, *p* < 0.05), FFA (r = 0.405, *p* < 0.05), and TAR (r = 0.326, *p* < 0.05). These results provide empirical support for the hypothesized relationships, confirming significant associations among the constructs and reinforcing the robustness of the proposed model.

### 4.4. Structural Equation Modeling Analysis

#### 4.4.1. Model Fit Assessment

**Figure 2:**
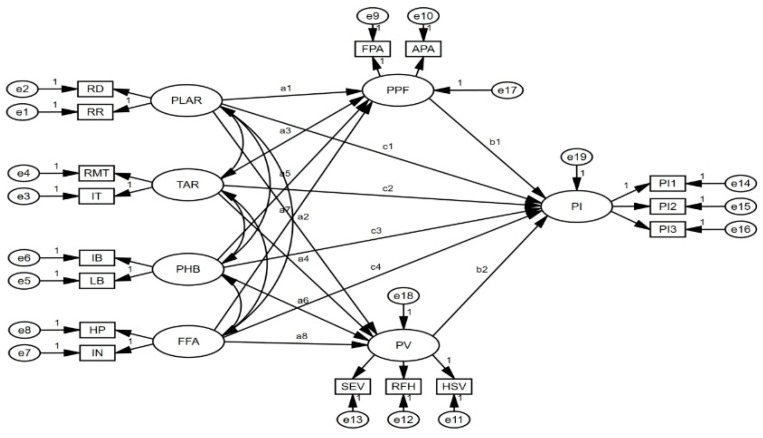
Structural equation model. Note: RR = Recommendation Relevance; RD = Recommendation Diversity; RMT = Recommendation Mechanism Transparency; IT = Information Transparency; IB = Immediate Health Benefits; LB = long-term health benefits; HP = Health Perception; IN = Ingredient Naturalness; FPA = Functional Packaging Attributes; APA = Aesthetic Packaging Appeal; SEV = Self-esteem Value; RFH = responsibility for health; HSV = Health and Safety Value; PI = purchase intention of functional foods; PLAR = AI recommendation personalization; TAR = AI recommendation transparency; PHB = perceived health benefits of functional foods; FFA = perceived naturalness of functional foods; PPF = perceived packaging of functional foods; PV = perceived value of functional foods. e1–e18, a1–a8, b1–b2, and c1–c4 represent measurement errors associated with the observed variables.

This study employed AMOS version 23 software to construct and analyze SEM. The model consists of seven latent variables and sixteen observed variables. To assess model fit, several commonly used fit indices were examined, including χ²/df, RMSEA, SRMR, CFI, and TLI. The results indicate that the model demonstrates an acceptable and strong fit with the data (χ²/df = 1.201, RMSEA = 0.022, SRMR = 0.027, CFI = 0.993, TLI = 0.99), all of which fall within the recommended thresholds [86,87]. These findings suggest that the model adequately represents the underlying structure and framework. Figure 2 shows the model.

#### 4.4.2. Path Analysis

The SEM analysis was conducted using AMOS 23 with the MLE method to examine the proposed hypotheses [86]. The path coefficients and their significance levels were evaluated to determine the strength and direction of the relationships among variables. Table 3 shows the path analysis and hypothesis testing results.

The results indicate that PLAR has a significant positive effect on PI (β = 0.195, *p* < 0.05), supporting H1a. However, TAR does not significantly influence PI (β = 0.037, *p* > 0.05), leading to the rejection of H1b. Among functional food attributes, PHB exhibits a significant positive effect on PI (β = 0.154, *p* < 0.05), supporting H1c, whereas perceived naturalness (FFA) is not significant (β = 0.032, *p* > 0.05), resulting in the rejection of H1d.

For the mediating variables, PLAR significantly influences PPF (β = 0.275, *p* < 0.001), supporting H2a, while TAR has no significant effect (β = 0.122, *p* > 0.05), rejecting H2b. Similarly, PHB positively influences PPF (β = 0.273, *p* < 0.001), supporting H2c, whereas FFA does not (β = 0.107, *p* > 0.05), rejecting H2d.

Regarding PV, all predictors show significant positive effects: PLAR (β = 0.343, *p* < 0.001), TAR (β = 0.144, *p* < 0.05), PHB (β = 0.201, *p* < 0.01), and FFA (β = 0.211, *p* < 0.01), supporting H3a, H3b, H3c, and H3d, respectively.

Finally, both mediating variables exhibit significant direct effects on PI. PPF positively influences PI (β = 0.200, *p* < 0.01), supporting H4a, while PV has a strong positive effect on PI (β = 0.326, *p* < 0.001), supporting H4b.

#### 4.4.3. Mediation Analysis

To examine the mediating roles of PPF and PV, the Bootstrap resampling method with 5000 iterations and a 95% CI was employed. A mediation effect is considered significant if the CI does not include zero. For this research, Table 4 shows the mediation analysis results.

For PLAR, the total effect on PI is significant (β = 0.362, 95% CI excluded 0). The direct effect remains significant (β = 0.195, *p* < 0.05), indicating a partial mediation. Specifically, both PLAR → PPF → PI (indirect effect = 0.055, 95% CI excluded 0) and PLAR → PV → PI (indirect effect = 0.112, 95% CI excluded 0) demonstrate significant mediation pathways, confirming that both perceived packaging and perceived value partially mediate the relationship between PLAR and PI.

For TAR, the total effect on PI is not significant (β = 0.108, 95% CI included 0), and the direct effect is also non-significant (β = 0.037, 95% CI included 0). The mediation analysis reveals that TAR → PPF → PI is not significant (indirect effect = 0.024, 95% CI included 0), whereas TAR → PV → PI exhibits a significant full mediation effect (indirect effect = 0.047, 95% CI excluded 0). These findings suggest that while TAR does not directly influence PI, it exerts an indirect effect through perceived value.

For perceived health benefits (PHB), the total effect on PI is significant (β = 0.275, 95% CI excluded 0), and the direct effect remains significant (β = 0.154, 95% CI excluded 0), indicating partial mediation. Both mediation pathways are significant, with PHB → PPF → PI (indirect effect = 0.055, 95% CI excluded 0) and PHB → PV → PI (indirect effect = 0.066, 95% CI excluded 0) confirming that perceived packaging and perceived value serve as partial mediators in the relationship between PHB and PI.

For perceived naturalness (FFA), the total effect on PI is not significant (β = 0.122, 95% CI included 0), and the direct effect is also non-significant (β = 0.032, 95% CI included 0). The mediation analysis indicates that FFA → PPF → PI does not exhibit a significant mediation effect (indirect effect = 0.021, 95% CI included 0). However, FFA → PV → PI demonstrates a significant full mediation effect (indirect effect = 0.069, 95% CI excluded 0), suggesting that perceived naturalness influences PI only through its impact on perceived value.

Overall, these findings underscore the critical mediating roles of perceived packaging and perceived value in shaping purchase intention. The presence of both partial and full mediation effects highlights the complexity of consumer decision-making processes, particularly in the context of AI-driven recommendations and functional food marketing.

## 5. Discussion

### 5.1. Summary and Interpretation of Key Findings

This study explores the impact of AI-driven recommendations and functional food attributes on consumer purchase intentions, incorporating the SOR framework. By examining AI recommendation personalization, AI recommendation transparency, perceived health benefits, and perceived naturalness, the research identifies their direct and indirect effects on purchase intention, with perceived packaging and perceived value serving as mediators. The findings provide empirical insights into how AI-driven personalization enhances consumer engagement, how transparency affects purchase behavior through value perception, and how functional food attributes operate through different cognitive pathways. These insights contribute to both theoretical advancements and practical applications in AI-assisted marketing and functional food branding. These findings underscore the importance of integrating both technological and product-related factors in understanding consumer behavior in health-oriented food contexts. Specifically, AI recommendation personalization was shown to directly enhance purchase intention, suggesting that tailored health-related product suggestions can effectively reduce decision complexity and increase consumer confidence in functional food choices. This supports previous findings that personalization improves engagement in digital environments but extends them to the domain of functional food purchasing, where health concerns are highly individualized [88]. In contrast, AI recommendation transparency indirectly affected purchase intention via perceived value, highlighting that while transparency strengthens trust and perceived fairness, it must translate into a clear consumer benefit to drive actual purchasing behavior.

Overall, these findings highlight that in the complex decision-making environment of functional food consumption, the interplay between AI recommendation characteristics and intrinsic product attributes jointly shapes consumer evaluations and behaviors. This comprehensive perspective advances previous research, which has often examined these elements separately, and demonstrates the necessity of considering how digital technologies like AI work in tandem with product-level factors to guide health-related food decisions in online settings [4,88]. These insights contribute to both theoretical advancements and practical applications in AI-assisted marketing and functional food branding.

### 5.2. Theoretical Implications

This study advances the theoretical understanding of AI-driven consumer behavior and functional food marketing by integrating the S-O-R framework to examine how AI recommendation characteristics and product attributes influence purchase intention. The findings highlight distinct mechanisms through which AI recommendation personalization, AI recommendation transparency, perceived health benefits, and perceived naturalness shape consumer decision-making, offering new insights into the role of AI-assisted marketing and cognitive processing in functional food choices.

First, this study contributes to the literature on AI-driven recommendations and consumer decision-making by revealing the differential effects of personalization and transparency. These two AI characteristics represent distinct psychological stimuli within the S-O-R framework, and their influence pathways shed light on how technology shapes health-related food choices. Specifically, personalization plays a direct and prominent role in enhancing purchase intention by creating a sense of individualized relevance, reducing decision fatigue, and fostering a stronger psychological connection between consumers and recommended products [89]. This insight expands previous personalization research by contextualizing its importance in functional food consumption, where health needs are highly specific and tailored recommendations may alleviate consumers’ cognitive load and health uncertainties [65].

Conversely, AI recommendation transparency does not directly influence purchase intention but has an indirect effect through perceived value. This aligns with existing studies on the “AI transparency paradox” [90], which suggest that while transparency increases trust, it does not always translate into action unless consumers perceive tangible value. This suggests that while understanding the rationale behind AI recommendations fosters trust and clarity, it does not independently drive purchasing behavior unless it translates into a stronger perception of value. Within the S-O-R framework, this study suggests that personalization serves as a more direct stimulus influencing both internal cognitive processing and behavior, whereas transparency requires an intermediary step through value perception to impact consumer decisions.

Beyond the technological factors, this study also contributes to functional food research by emphasizing the differentiated roles of health benefits and naturalness as product attributes. The findings confirm that clear, credible health benefits are a dominant motivator in functional food purchases, reinforcing prior studies highlighting the importance of explicit health claims [91]. However, the indirect effect of naturalness through perceived value reveals that, while naturalness is an attractive quality, it must be meaningfully translated into tangible consumer benefits such as safety, purity, and authenticity before influencing behavior. This extends the existing literature on the value-attitude-behavior hierarchy [92], suggesting that intrinsic product attributes require cognitive validation to impact purchasing decisions.

Moreover, by demonstrating the mediating roles of perceived packaging and perceived value, this study clarifies how external stimuli translate into consumer behavior through internal evaluations. The finding that packaging mediates the effects of personalization and health benefits reinforces the concept that packaging serves as a key communicative vehicle, especially in conveying personalized and health-centric messages. Meanwhile, perceived value emerges as a comprehensive mediator across all predictor variables, highlighting its centrality in health-driven food consumption and the psychological trade-offs consumers make between benefits and costs.

Overall, this study contributes to a more integrated understanding of AI-driven health food marketing by showing how technological features and product attributes jointly influence consumer decision-making. This study extends research on AI transparency by demonstrating its indirect effect through perceived value, challenges assumptions about the direct impact of naturalness on purchase behavior, and reinforces the central role of perceived value as a mediating mechanism. This integrated perspective provides a richer theoretical foundation for future studies exploring the intersection of digital personalization, consumer trust, and health-oriented consumption.

### 5.3. Managerial Implications

The findings of this study provide practical insights for optimizing AI-driven recommendation systems and enhancing marketing strategies for functional foods. Firstly, AI-driven recommendations should be designed to enhance both personalization and transparency but with distinct strategic goals tailored to functional foods. Personalized recommendations should move beyond generic product suggestions and incorporate consumer dietary habits, specific health goals, and past interactions with functional food categories. For instance, consumers actively seeking immune-boosting functional foods should receive recommendations highlighting scientifically validated benefits of probiotics or vitamin-enriched products. Since personalization directly influences purchase intention and enhances consumer perceptions of packaging and value, AI recommendation systems should integrate health-focused content, such as clinical research summaries, functional ingredient breakdowns, and expert endorsements. This strategic approach leverages the psychological principle of relevance, whereby consumers perceive higher value in information that aligns with their personal health needs [93,94] and can strengthen engagement in health-related food decisions. Additionally, personalization can reduce cognitive overload in functional food choices by simplifying complex nutritional information, which is particularly important in categories with diverse health claims and formulations [71]. By reducing decision fatigue and enhancing product relevance, well-designed AI personalization not only improves purchase intention but also fosters long-term user satisfaction with the recommendation system.

Moreover, AI recommendation transparency should be positioned as a tool to reduce uncertainty and build trust in functional food claims rather than a direct purchase driver. Given the complex health claims often associated with functional foods, consumers may hesitate due to skepticism regarding efficacy and safety. Transparent AI recommendations should explain why a product is suggested, incorporating scientific validation, dietary compatibility, and third-party certifications. For example, when an AI system recommends an omega-3-enriched functional food, it should also provide supporting evidence, such as certifications from nutritional organizations or clinical research findings on heart health benefits. This approach ensures that transparency strengthens product credibility rather than merely disclosing recommendation mechanisms without adding value. Beyond trust-building, transparency also enhances consumers’ perceived fairness and control over the decision-making process [65]. Providing insight into how recommendations are generated can mitigate concerns about manipulation or bias, which is particularly relevant in health-related contexts where data privacy and accuracy are critical considerations [95]. These mechanisms not only foster acceptance of AI recommendations but also indirectly enhance perceptions of product value and safety.

Secondly, functional food brands should refine their packaging and communication strategies to effectively convey health benefits and naturalness, leveraging insights from consumer perception. Since perceived health benefits have both direct and indirect effects on purchase intention, packaging should prominently feature clinically validated claims, ingredient efficacy, and structured nutrition labeling. For example, products marketed for cognitive health enhancement (e.g., omega-3-fortified beverages) should highlight scientific studies on brain function directly on the packaging, making critical information more accessible and reassuring to consumers.

On the other hand, since perceived naturalness does not directly drive purchase intention but influences consumer behavior through perceived value, brands must focus on substantiating their natural positioning rather than simply labeling products as “natural”. Instead of vague claims like “100% natural”, packaging should include clear ingredient sourcing details, minimal processing information, and sustainability credentials. For instance, a plant-based functional protein bar should reinforce its natural attributes with visual storytelling about raw ingredient origins, the absence of artificial additives, and eco-friendly packaging materials. Additionally, AI recommendations can support this by offering contextualized explanations, such as ingredient traceability data, organic certification details, or clean-label benefits, to further strengthen consumer confidence in product value.

Incorporating these strategies acknowledges the growing consumer demand for transparency, authenticity, and sustainability in health-related purchases [96], particularly as consumers become more informed and critical of health claims in the functional food market [15]. Combining AI-driven insights with packaging communication creates a cohesive, trustworthy narrative that bridges the gap between personalized recommendations and tangible product attributes.

By aligning AI-driven personalization with consumer health priorities and leveraging packaging to reinforce functional benefits and naturalness, businesses can build trust, enhance product differentiation, and drive greater consumer engagement in the functional food market. These strategies ensure that AI technology is not merely a sales tool but an educational and empowerment mechanism, bridging the gap between scientific evidence and consumer purchase decisions in the evolving functional food landscape.

### 5.4. Limitations and Future Research Directions

While this study provides valuable insights into how AI-driven recommendation systems and functional food attributes influence consumer purchase intentions, several areas warrant further exploration. First, this study employs a questionnaire-based SEM approach, which effectively captures consumers’ subjective perceptions and purchase intentions while offering robust analytical power to examine complex relationships [92]. However, SEM primarily establishes associations rather than definitive causal relationships, as it relies on cross-sectional data. Experimental studies that systematically manipulate AI recommendation characteristics and functional food attributes under controlled conditions could provide stronger causal validation. Future research could adopt randomized controlled experiments or A/B testing methodologies to investigate how variations in AI-driven personalization, transparency, and functional food labeling influence consumer decision-making in real-time purchasing scenarios. Moreover, future longitudinal research could further explore the long-term impacts of AI-driven recommendations on consumer behavior. For example, repeated exposure to AI-generated suggestions may foster sustained trust and encourage brand loyalty, but it may also lead to AI fatigue, wherein consumers become less responsive or even resistant to algorithmic recommendations over time. While previous studies have applied longitudinal data to examine AI applications in contexts such as electronic health records [96], these long-term behavioral effects in consumer decision-making remain insufficiently explored. Investigating these temporal dynamics is essential to understanding the enduring influence of AI systems on consumer engagement and purchase behaviors.

Second, the data were collected from consumers within a single-country context, ensuring cultural consistency but potentially limiting the generalizability of findings across different cultural and economic environments. Consumer attitudes toward AI technology, functional foods, and digital recommendations vary significantly across cultures, influenced by factors such as health consciousness, technology adoption readiness, and trust in AI-driven decision-making [97]. Future research could adopt cross-cultural comparative studies to examine how consumer responses to AI-driven recommendations differ based on cultural and regulatory contexts. Leveraging established cultural dimensions frameworks, such as Hofstede’s model, would enable deeper insights into the global applicability of AI-driven marketing strategies in the functional food sector.

Third, this study examines the direct and mediating effects of AI recommendation characteristics and functional food attributes on purchase intention. However, potential moderating factors were not explicitly tested. It does not account for individual differences and other contextual factors that may influence how consumers process AI-driven recommendations and product information. Previous studies suggest that demographic factors such as age and gender can significantly shape consumer responses to products, including purchase intentions. Additionally, recent research highlights the importance of familiarity with technology, and consumers with higher digital literacy may demonstrate greater trust and responsiveness to AI-generated recommendations. Future research could explore these moderating effects through segmentation or multi-group analyses to examine whether the relationships identified in this study differ across diverse demographic and psychographic profiles. Such investigations would provide valuable insights into whether AI-driven functional food marketing strategies are equally effective across varying consumer characteristics and situational contexts [98].

Additionally, while this study focuses on the positive effects of AI-driven recommendations in enhancing perceived value and packaging evaluation, it is important to acknowledge that some consumers may approach algorithmic suggestions with skepticism, viewing them as potentially intrusive or overly persuasive [99]. Recent studies indicate that although AI models may outperform humans in certain tasks across various industries, concerns about potential risks, data privacy, and ethical issues can significantly influence consumers’ willingness to trust AI systems [100,101]. Furthermore, research on personalized recommendations highlights the potential risk of information cocoons, where consumers are repeatedly exposed to narrow, homogeneous information, potentially limiting their ability to make well-rounded and informed decisions [1]. Future research should explore how factors such as trust in AI, concerns about data privacy, and perceptions of algorithmic fairness shape consumer responses to AI-driven functional food recommendations. Examining how these concerns interact with perceived value and packaging evaluation could provide deeper insights into the psychological processes driving consumer decisions. Moreover, such investigations would be particularly valuable in markets with varying levels of regulatory oversight and consumer protection related to health claims, where sensitivity to ethical considerations and transparency may differ.

Finally, this study primarily focuses on the cognitive and evaluative aspects of AI-driven recommendations and functional food attributes, and it is important to acknowledge the role of sensory quality and product-elicited emotions in shaping consumer purchase decisions. Sensory factors such as taste, texture, and aroma are fundamental to functional food acceptance and long-term consumer retention [102]. Moreover, emotions elicited by product presentation, AI-driven recommendations, and packaging design can significantly influence purchasing behavior beyond rational assessments of health benefits and naturalness [103]. Future research could explore how AI personalization and transparency interact with sensory perceptions and emotional responses to further shape consumer attitudes and purchase decisions. Investigating these factors would provide a more holistic understanding of functional food consumer behavior in AI-assisted marketing contexts.

## Figures and Tables

**Table 1:** Reliability and convergent validity of measurement constructs.

Constructs	Items	AVE	CR	Cronbach’s α
Personalization of AI Recommendations	Recommendation Relevance	0.618	0.829	0.823
	Recommendation Diversity	0.58	0.805	0.805
Transparency of AI Recommendations	Information Transparency	0.551	0.786	0.779
	Recommendation Mechanism Transparency	0.492	0.744	0.743
Perceived Health Benefits of Functional Foods	Immediate Health Benefits	0.59	0.812	0.805
	Long-term Health Benefits	0.532	0.773	0.774
Functional Food Attributes	Ingredient Naturalness	0.604	0.82	0.812
	Health Perception	0.558	0.791	0.791
Perceived Packaging of Functional Foods	Functional Packaging Attributes	0.558	0.791	0.783
	Aesthetic Packaging Appeal	0.574	0.802	0.801
Perceived Value of Functional Foods	Health and Safety Value	0.551	0.786	0.781
	Responsibility for Health	0.596	0.816	0.815
	Self-esteem Value	0.607	0.822	0.822
Purchase Intention	Purchase Intention	0.535	0.774	0.769

Note: AVE = Average Variance Extracted; CR = Composite Reliability.

**Table 2:** Pearson’s correlation matrix.

Variables	Mean	Standard Deviation	PLAR	TAR	PHB	FFA	PPF	PV	PI
PLAR	4.541	1.263	1						
TAR	4.531	1.127	0.218 **	1					
PHB	4.742	1.232	0.344 **	0.319 **	1				
FFA	4.493	1.240	0.367 **	0.349 **	0.310 **	1			
PPF	4.621	1.197	0.365 **	0.269 **	0.376 **	0.311 **	1		
PV	4.602	1.135	0.439 **	0.326 **	0.404 **	0.405 **	0.387 **	1	
PI	4.996	1.315	0.447 **	0.282 **	0.424 **	0.354 **	0.422 **	0.507 **	1

Note: ** *p* < 0.01; PLAR = AI recommendation personalization; TAR = AI recommendation transparency; PHB = perceived health benefits of functional foods; FFA = perceived naturalness of functional foods; PPF = perceived packaging of functional foods; PV = perceived value of functional Foods; PI = purchase intention of functional foods.

**Table 3:** Path analysis and hypothesis testing results.

Hypothesis	Path			β	SE	t	*p*	Decision
H1a	PLAR	→	PI	0.195	0.088	2.596	0.009	Accepted
H1b	TAR	→	PI	0.037	0.084	0.561	0.574	Rejected
H1c	PHB	→	PI	0.154	0.083	2.274	0.023	Accepted
H1d	FFA	→	PI	0.032	0.083	0.462	0.644	Rejected
H2a	PLAR	→	PPF	0.275	0.066	3.778	***	Accepted
H2b	TAR	→	PPF	0.122	0.072	1.713	0.087	Rejected
H2c	PHB	→	PPF	0.273	0.068	3.836	***	Accepted
H2d	FFA	→	PPF	0.107	0.07	1.451	0.147	Rejected
H3a	PLAR	→	PV	0.343	0.061	4.973	***	Accepted
H3b	TAR	→	PV	0.144	0.065	2.185	0.029	Accepted
H3c	PHB	→	PV	0.201	0.061	3.103	0.002	Accepted
H3d	FFA	→	PV	0.211	0.064	3.059	0.002	Accepted
H4a	PPF	→	PI	0.2	0.09	2.844	0.004	Accepted
H4b	PV	→	PI	0.326	0.104	4.099	***	Accepted

Note: *** *p* < 0.001. Abbreviations are consistent with those defined in Table 2.

**Table 4:** Mediation analysis results.

Path	Estimate	SE	Lower	Upper	*p*
PLAR → PI (TE)	0.362	0.068	0.226	0.492	0.001
PLAR → PI (DE)	0.195	0.074	0.049	0.337	0.011
PLAR → PI (IE)	0.167	0.042	0.095	0.262	0.000
PLAR → PPF → PI	0.055	0.024	0.017	0.112	0.004
PLAR → PV → PI	0.112	0.034	0.057	0.192	0.000
TAR → PI (TE)	0.108	0.065	−0.026	0.23	0.112
TAR → PI (DE)	0.037	0.065	−0.093	0.158	0.63
TAR → PI (IE)	0.071	0.035	0.013	0.155	0.018
TAR → PPF → PI	0.024	0.018	−0.001	0.074	0.056
TAR → PV → PI	0.047	0.026	0.005	0.11	0.025
PHB → PI (TE)	0.275	0.068	0.139	0.404	0.001
PHB → PI (DE)	0.154	0.068	0.023	0.288	0.021
PHB → PI (IE)	0.12	0.04	0.053	0.214	0.001
PHB → PPF → PI	0.055	0.025	0.017	0.122	0.003
PHB → PV → PI	0.066	0.029	0.019	0.132	0.008
FFA → PI (TE)	0.122	0.071	−0.019	0.259	0.083
FFA → PI (DE)	0.032	0.068	−0.102	0.165	0.625
FFA → PI (IE)	0.09	0.037	0.027	0.176	0.006
FFA → PPF → PI	0.021	0.018	−0.005	0.071	0.099
FFA → PV → PI	0.069	0.029	0.022	0.141	0.005

Note: Abbreviations are consistent with those defined in Table 1. Additional abbreviations used in this table are listed below: TE = total effect; DE = direct effect; IE = indirect effect.

## Data Availability

The original contributions presented in this study are included in the article. Further inquiries can be directed to the corresponding authors.

## Abbreviations

AI	Artificial Intelligence
TAM	Technology Acceptance Model
AVE	Average Variance Extracted
CFA	Confirmatory Factor Analysis
CR	Composite Reliability
EFA	Exploratory Factor Analysis
MLE	Maximum Likelihood Estimation
PI	Purchase Intention of Functional Foods
PLAR	AI Recommendation Personalization
TAR	AI Recommendation Transparency
PHB	Perceived Health Benefits of Functional Foods
FFA	Perceived Naturalness of Functional Foods
PPF	Perceived Packaging of Functional Foods
PV	Perceived Value of Functional Foods
SOR	Stimulus–Organism–Response
RR	Recommendation Relevance
RD	Recommendation Diversity
RMT	Recommendation Mechanism Transparency
IT	Information Transparency
IB	Immediate Health Benefits
LB	Long-term Health Benefits
HP	Health Perception
IN	Ingredient Naturalness
FPA	Functional Packaging Attributes
APA	Aesthetic Packaging Appeal
SEV	Self-esteem Value
RFH	Responsibility for Health
HSV	Health and Safety Value
